# Functional Characterization of the Plasmacytoma Variant Translocation 1 Gene (PVT1) in Diabetic Nephropathy

**DOI:** 10.1371/journal.pone.0018671

**Published:** 2011-04-22

**Authors:** M. Lucrecia Alvarez, Johanna K. DiStefano

**Affiliations:** Diabetes, Cardiovascular and Metabolic Diseases Center, Translational Genomics Research Institute, Phoenix, Arizona, United States of America; University of Hong Kong, Hong Kong

## Abstract

We previously observed association between variants in the plasmacytoma variant translocation 1 gene (*PVT1*) and end-stage renal disease (ESRD) attributed to both type 1 and type 2 diabetes, and demonstrated *PVT1* expression in a variety of renal cell types. While these findings suggest a role for *PVT1* in the development of ESRD, potential mechanisms for involvement remain unknown. The goal of this study was to identify possible molecular mechanisms by which *PVT1* may contribute to the development and progression of diabetic kidney disease. We knocked-down *PVT1* expression in mesangial cells using RNA interference, and analyzed RNA and protein levels of fibronectin 1 (*FN1*), collagen, type IV, alpha 1 (*COL4A1*), transforming growth factor beta 1 (*TGFB1*) and plasminogen activator inhibitor-1 (*SERPINE1 or PAI-1*) by qPCR and ELISA, respectively. *PVT1* expression was significantly upregulated by glucose treatment in human mesangial cells, as were levels of FN1, COL4A1, TGFB1, and PAI-1. Importantly, *PVT1* knockdown significantly reduced mRNA and protein levels of the major ECM proteins, FN1 and COL4A1, and two key regulators of ECM proteins, TGFB1 and PAI-1. However, we observed a higher and more rapid reduction in levels of secreted FN1, COL4A1, and PAI-1 compared with TGFB1, suggesting that at least some of the *PVT1* effects on ECM proteins may be independent of this cytokine. These results indicate that *PVT1* may mediate the development and progression of diabetic nephropathy through mechanisms involving ECM accumulation.

## Introduction

Diabetic nephropathy is the most common cause of chronic renal failure in developed countries and accounts for most of the reduced life expectancy in individuals with diabetes [1]. The prevalence of ESRD attributed to diabetes is increasing, mainly due to the rising prevalence of type 2 diabetes mellitus (T2D), and the decreasing age of T2D onset [1], [2]. Despite the growing magnitude of the disease, the molecular mechanisms underlying the etiology of diabetic nephropathy remain poorly understood. Risk factors for diabetic nephropathy include duration of diabetes, glycemic control, hypertension, and hyperlipidemia [3], [4]; however, genetic factors are also strong determinants of disease risk [5], [6], [7]. Correspondingly, results from genome-wide association studies to identify loci for diabetic kidney disease are now becoming available in the literature [8], [9], [10], [11], but at the present time, the clinical relevance of these, and other genetic findings, awaits additional validation and functional characterization to identify the molecular mechanisms by which these genes impact upon disease pathophysiology.

We previously utilized a genome-wide SNP genotyping approach to identify loci underlying susceptibility to ESRD attributed to T2D, and found the strongest evidence for association with variants in the gene encoding plasmacytoma variant translocation 1 or *PVT1* [9]. We later validated this locus in a replication study comprised of individuals with ESRD attributed to T1D, and demonstrated *PVT1* expression in a number of diverse renal cell types [12]. *PVT1* is located on 8q24, and in humans, is well known for its participation in recurrent translocations between this region and chromosomes 2 and 22 [13], [14]. The first exon of the gene is co-amplified with MYC in colon carcinoma cell lines [15], and overexpression of *PVT1* also contributes to ovarian and breast cancer [16]. Although the *PVT1* locus encodes a number of alternative transcripts, no *PVT1* protein product has yet been identified. Instead, *PVT1* likely represents a non-coding RNA that, when amplified and over-expressed, increases cell proliferation and inhibits apoptosis [16].

While the relationship between *PVT1* and certain forms of cancer has been firmly established, the role that this gene may play in mediating the development of kidney disease in diabetes is presently not known. However, it is well-recognized that excessive accumulation of extracellular matrix in the glomeruli is a hallmark of diabetic nephropathy. Mesangial cells (MC) play a central role in the development of diabetic nephropathy because they regulate glomerular filtration rate (GFR) through their contractility [17], [18] and produce the ECM proteins that accumulate in the glomerular mesangium of patients with diabetic nephropathy [19], [20]. Mesangial expansion impinges on glomerular capillaries, reducing the surface available for filtration and narrowing or occluding the lumen, and it is widely held that these mesangial changes are one of the main causes of declining renal function in diabetic nephropathy [21]. In light of this background, we decided to initiate the functional characterization of *PVT1* in the kidney by first investigating its role in mesangial cells.

Based on the findings of association between variants in *PVT1* and diabetic ESRD, and expression in different cells of the kidney, we sought to obtain biological evidence to support a role for this gene in the disease process. The goal of this study, therefore, was to identify possible molecular mechanisms by which *PVT1* may contribute to the development and progression of diabetic nephropathy in mesangial cells. We first determined the extent to which *PVT1* expression is regulated by glucose in the kidney, and because diabetic nephropathy is characterized by excessive accumulation of extracellular matrix (ECM) in the glomeruli, we also assessed the effect of glucose on mRNA and protein expression of specific ECM components in relation to *PVT1* in mesangial cells. Finally, as a first step toward characterizing the role of *PVT1* in the accumulation of ECM proteins in MC, we examined the effect of *PVT1* knockdown using RNA interference on mRNA and protein expression of these components under high glucose conditions.

## Results

### Effect of glucose on expression of PVT1 and ECM-related proteins

**Figure 1:**
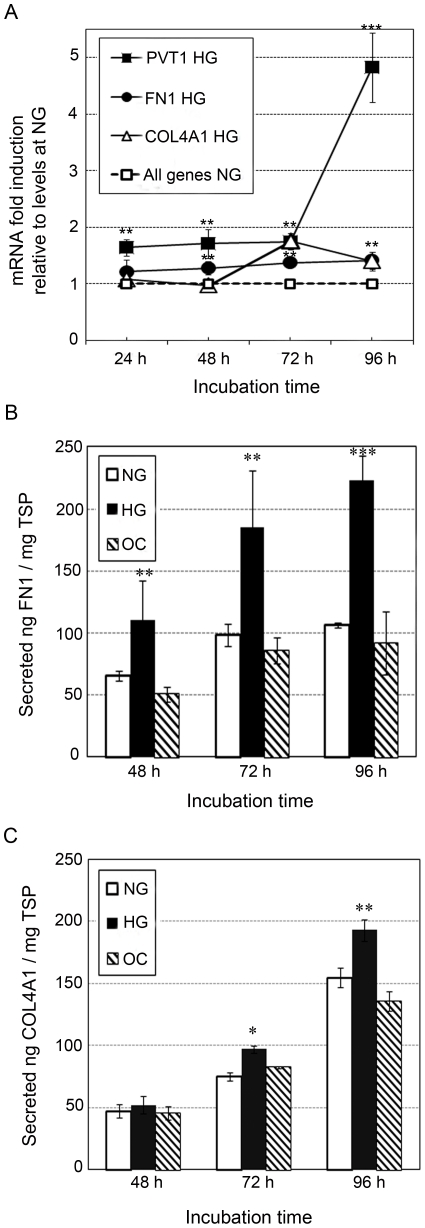
Effect of glucose on expression of *PVT1*, *FN1*, and *COL4A1* in normal human mesangial cells (MC). Prior to treatment with high glucose, MC, at approximately 70% confluence, were cultured in serum-free MsBM medium for 24 hours to arrest and synchronize cell growth. After this time period, MC were grown for 24, 48, 72 or 96 h in MsBM medium supplemented with 5% FBS, containing either normal glucose (NG; 5.6 mM), NG+3-O-methyl-D-glucose (3-O-MG) to control for osmotic effects (OC: 5.6 mM glucose+24.4 mM 3-O-MG) or high glucose (HG: 30 mM). (**A**) Relative quantification of *PVT1*, *FN1* and *COL4A1* mRNA by TaqMan qPCR. The mRNA level for all the genes was arbitrary set to 1 under NG conditions at each time point. Data are presented as mRNA fold-increase incubated under HG or NG conditions. Quantification of secreted FN1 (**B**) and COL4A1 (**C**) proteins by ELISA. Data are expressed as nanograms of FN1 or COL4A1 per total soluble protein (TSP). Results represent average of three independent experiments. Data are means ± SD. A.U.: arbitrary units. * *P*<0.05; ** *P*<0.01; *** *P*<0.001 with respect to NG at each time point.

We first investigated the effect of glucose on the expression of *PVT1* in (MC). As shown in Fig. 1A, *PVT1* expression levels in MC were increased at all time points under high glucose (HG: 30 mM) compared to normal glucose (NG: 5.6 mM) or 3-O-MG (OC: 5.6 mM glucose+24.4 mM 3-O-MG) conditions; the highest increase was seen at 96 hours (Fig. 1A). Because diabetic nephropathy is characterized by excessive accumulation of ECM in the glomeruli, we also assessed the effect of glucose on mRNA expression of the two main components of ECM in MC: FN1 and COL4A1, and found increases of *FN1* expression at 48 h, 72 h, and 96 h, but not at 24 h. *COL4A1* mRNA levels were also significantly higher under HG versus NG or OC conditions at 72 h and 96 h, but not at 24 h or 48 h (Fig. 1A). The mRNA levels for all the analyzed ECM-related genes expressed in MC under NG conditions for 0 h (basal conditions) were used as calibrator in the qPCR analysis and arbitrarily set to 1. We observed that the ECM-related mRNA levels in MC under NG for 24 h, 48 h, 72 h and 96 h are not significantly different from NG at 0 h (data not shown). In Fig. 1A, we arbitrarily set to 1 the mRNA levels for all the ECM-related genes analyzed in MC under NG conditions for 0 h, 24 h, 48 h, 72 h and 96 h.

We next quantified FN1 and COL4A1 protein concentrations in media from MC cultured for 48 h, 72 h or 96 h under NG, HG, or OC conditions (*PVT1* is a non-coding RNA and, therefore, has no protein product to quantify). As shown in Fig. 1B, the FN1 secreted by MC in HG conditions progressively increased from 70% higher than NG or OC at 48 h to 110% higher than NG or OC at 96 h of incubation, in agreement with the progressive increase of FN1 mRNA from 30% to 40% at 48 h and 96 h, respectively (Fig. 1A). The increase of secreted COL4A1 was 30% and 25% higher in MC under HG compared to NG or OC conditions at 72 h and 96 h of incubation, respectively. However, we did not find a significant difference (*P*>0.05) between secreted COL4A1 in HG versus NG or OC conditions at 48 h (Fig. 1C), which is concordant with levels of *COL4A1* mRNA incubated in HG vs. NG or OC for 24 h and 48 h (Fig. 1A).

**Figure 2:**
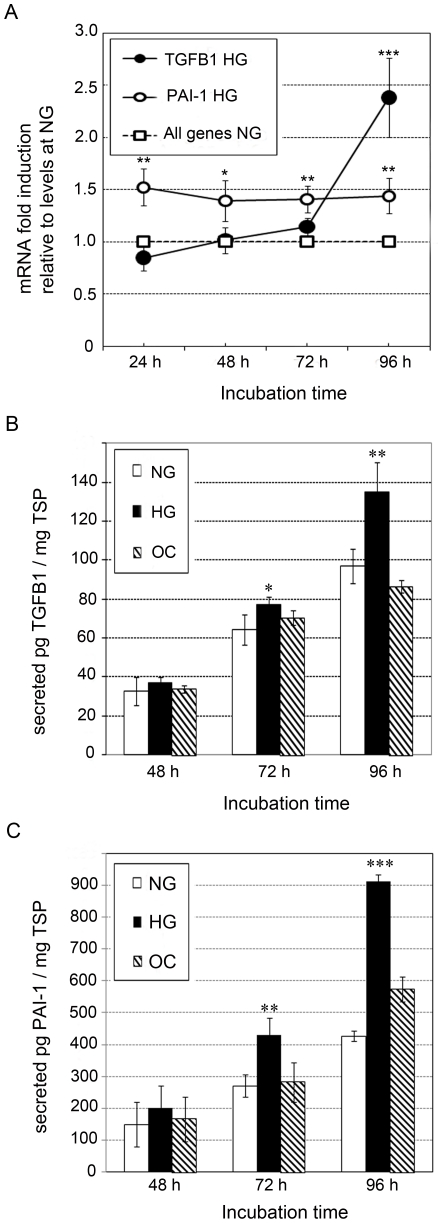
Effect of glucose on TGFB1 and PAI-1 expression in MC. (**A**) Relative quantification of *TGFB1* and *PAI-1* mRNA by TaqMan qPCR. The mRNA level for all the genes was arbitrary set to 1 under NG conditions at each time point. Data are presented as mRNA fold-increase in MC incubated in HG or NG OC conditions. Quantification of secreted TGFB1 (**B**) and PAI-1 (**C**) proteins by sandwich ELISA. Data are expressed as picograms of PAI-1 or TGFB1 per total soluble protein (TSP). Results represent average of three independent experiments. Data are means ± SD. A.U.: arbitrary units. * *P*<0.05; ** *P*<0.01; *** *P*<0.001 with respect to NG at each time point.

Because TGFB1 is a key mediator of ECM accumulation in diabetic nephropathy, and PAI-1 inhibits ECM turnover, we also examined the effect of glucose on expression of these genes. As shown in Fig. 2A, *TGFB1* mRNA expression was 0.15-fold and 1.35-fold higher under HG versus NG conditions at 72 h and 96 h, respectively. *PAI-1* mRNA expression was increased approximately 0.5-fold under HG versus NG conditions at all time points (Fig. 2A). The mRNA levels for all the analyzed ECM-related genes expressed in MC under NG conditions for 0 h (basal conditions) were used as calibrator in the qPCR analysis and arbitrarily set to 1. We observed that the ECM-related mRNA levels in MC under NG for 24 h, 48 h, 72 h and 96 h are not significantly different from NG at 0 h (data not shown). In Fig. 2A, we arbitrarily set to 1 the mRNA levels for all the ECM-related genes analyzed in MC under NG conditions for 0 h, 24 h, 48 h, 72 h and 96 h. We next quantified levels of secreted TGFB1 and PAI-1 protein in media from MC cultured for 48, 72 or 96 h under NG, HG, or OC conditions. Levels of secreted TGFB1 were 10%, 20% and 40% higher under HG versus NG or OC conditions at 48 h, 72 h, and 96 h, respectively (Fig. 2B). Levels of secreted PAI-1 were 35%, 50% and 115% higher under HG compared to NG or OC conditions at the same time points (Fig. 2C).

### Effect of PVT1 knockdown on mRNA and protein expression of FN1 and COL4A1

**Figure 3:**
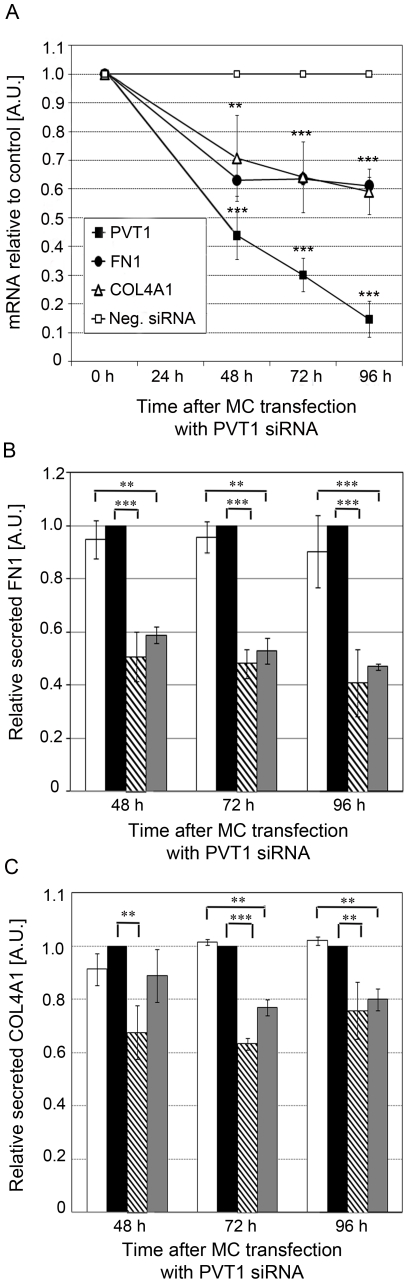
Effect of *PVT1* knockdown on the expression of FN1 and *COL4A1*. (**A**) *PVT1*, *FN1* and *COL4A1* mRNA levels following transfection of MC with *PVT1* siRNA. Data were obtained by qPCR analysis and presented as mRNA fold-increase compared with Neg siRNA. Non-transfected (NT) MC were used as a second negative control. Quantification of secreted FN1 (**B**) and COL4A1 (**C**) in media from MC. Data were obtained by sandwich ELISA and compared with Neg siRNA. White and grey bars represent non-transfected (NT) MC under HG and NG conditions, respectively; black and striped bars represent MC under HG conditions transfected with Neg or *PVT1* siRNA, respectively. Results represent average from three independent experiments. Data are means ± SD. A.U.: arbitrary units. * *P*<0.05; ** *P*<0.01; *** *P*<0.001.

To determine the role of *PVT1* in the accumulation of ECM proteins in MC, we depleted *PVT1* gene expression using RNA interference techniques under HG conditions and quantified levels of *PVT1*, *FN1* and *COL4A1* mRNA levels by qPCR. We observed a 0.6-fold, 0.7-fold and 0.85-fold decrease in *PVT1* mRNA levels in MC transfected with *PVT1* siRNAs compared to those transfected with Neg siRNA at 48 h, 72 h and 96 h post-transfection, respectively. In addition, siRNA knockdown of *PVT1* expression was accompanied by a 0.3 and 0.4-fold decrease of *FN1* and *COL4A1* mRNA expression, respectively (Fig. 3A). Correspondingly, levels of secreted FN1 (Fig. 3B) and COL4A1 (Fig. 3C) were also significantly reduced after *PVT1* knockdown in MC under HG conditions, to the basal levels found in MC under NG conditions (grey bars).

### Effect of PVT1 knockdown on mRNA and protein expression of TGFB1 and PAI-1

**Figure 4:**
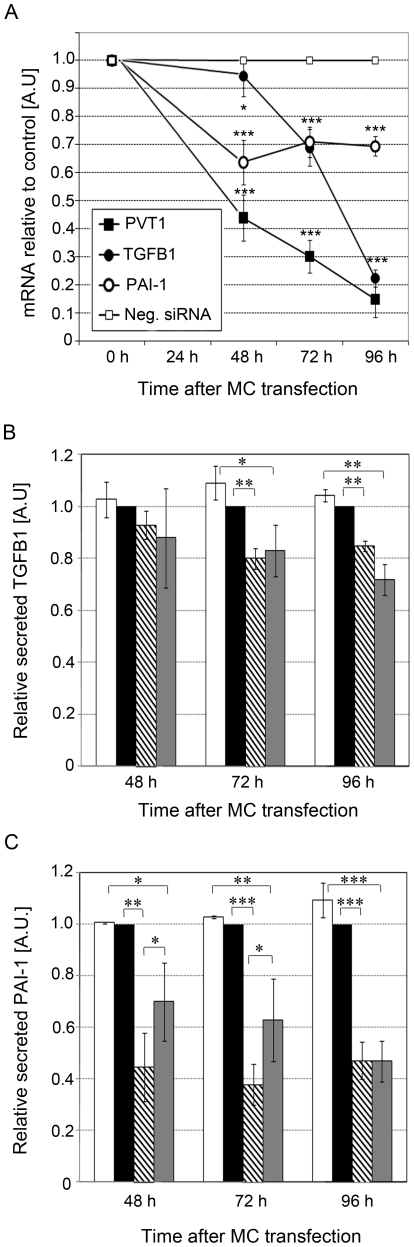
Effect of *PVT1* gene knockdown on TGFB1 and PAI-1. (**A**) *PVT1*, *TGFB1* and *PAI-1* mRNA levels following MC transfection with *PVT1* siRNA. Data were obtained by qPCR analysis and presented as mRNA fold-increase compared with Neg siRNA. Non-transfected (NT) MC were used as a second negative control. Quantification of secreted TGFB1 (**B**) and PAI-1 (C) in media from MC after cell transfection with *PVT1* siRNAs. Data were obtained by sandwich ELISA and compared with Neg siRNA. White and grey bars represent non-transfected (NT) MC under HG and NG conditions; black and striped bars, MC under HG conditions transfected with Neg or *PVT1* siRNA, respectively. Results represent the average of three independent experiments. Data are means ± SD. A.U.: arbitrary units. * *P*<0.05; ** *P*<0.01; *** *P*<0.001.

We also analyzed *TGFB1* and *PAI-1* mRNA levels by qPCR and their secreted protein products by ELISA in MC transfected with *PVT1* siRNAs. As shown in Fig. 4A, levels of *TGFB1* mRNA were decreased 0.3-fold and 0.8-fold in MC transfected with *PVT1* siRNAs at 72 h, and 96 h compared to cells transfected with Neg siRNA or non-transfected cells (Fig. 4A). Levels of secreted TGFB1 were decreased by 20%, and 15% at the same time points, respectively (Fig. 4B). We observed a much greater 30–35% decrease in mRNA (Fig. 4A) and 50–60% decrease in secreted protein (Fig. 4C) levels of PAI-1 in MC transfected with *PVT1* siRNA compared to Neg siRNA or non-transfected cells. The levels of secreted TGFB1 (Fig. 4B) and PAI-1 (Fig. 4C) were significantly reduced after *PVT1* knockdown in MC under HG conditions, to the basal levels found in MC under NG conditions.

## Discussion

We first identified *PVT1* as a candidate gene for ESRD using a genome-wide SNP association study in American Indians with T2D [9], and later validated this locus in an independent group of individuals with T1D [12]. Findings of expression in different cell types of the kidney provided preliminary evidence that *PVT1* may influence metabolic dysregulation in this tissue preceding the development of renal failure in diabetes. Here, we provide additional biological evidence supporting a role for *PVT1* in the pathogenesis of diabetic nephropathy.

In the current study, we found that *PVT1* expression levels increase up to 5-fold in response to hyperglycemic conditions, showing an effect of glucose on *PVT1* regulation. Reduction of *PVT1* expression by siRNA significantly affected mRNA and protein levels of all ECM components examined, suggesting that *PVT1* contributes to ECM deposition in the glomeruli, one of the major pathological features of diabetic nephropathy.

**Figure 5:**
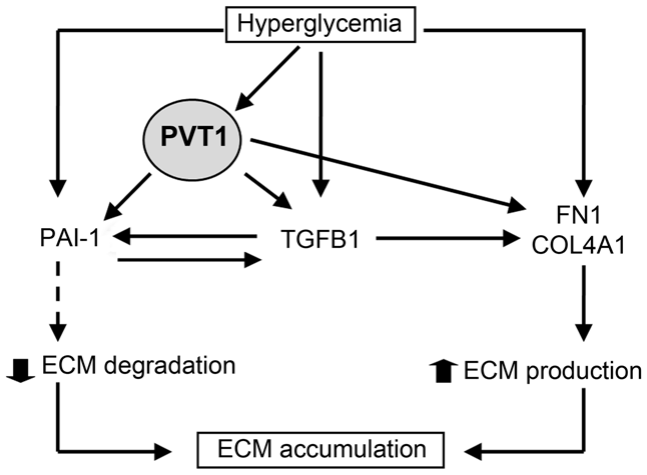
Potential mechanism for *PVT1* involvement in ECM deposition. Hyperglycemia causes an excessive accumulation of extracellular matrix (ECM) in the glomerular mesangium which constitutes the major pathological feature of the glomerulosclerosis or glomerular fibrosis. Hyperglycemic conditions induce an increase in *PVT1* expression in MC which contributes to the increase of the two main ECM components, FN1 and COL4A1, as well as the two main regulators of ECM accumulation in the glomerule, PAI-1 and TGFB1. PAI-1 is the main inhibitor of glomerular ECM degradation and TGFB1 promotes glomerular fibrosis by upregulating genes encoding ECM proteins and PAI-1. PAI-1 and TGFB1 transcriptionally regulate each other creating a vicious cycle of reciprocal stimulation that perpetuates the fibrotic response. *PVT1* may contribute to ECM accumulation mainly through a TGFB1-independent mechanism (see text). FN1: fibronectin 1; COL4A1: alpha 1, type IV, collagen; PAI-1: plasminogen activator inhibitor; TGFB1: transforming growth factor beta 1.

One of the more important findings of this work was that *PVT1* effects on expression of FN1, COL4A1, and PAI-1 may be mediated independently of TGFB1, a well-known profibrotic factor which promotes tissue fibrosis by upregulating genes encoding ECM proteins in response to hyperglycemia [20]. In our study, we found a higher and more rapid decrease in FN1, COL4A1 and PAI-1 compared to TGFB1 in response to *PVT1* knockdown. Specifically, at 48 hours following *PVT1* knockdown, no significant effect on secreted TGFB1 was apparent, yet levels of secreted FN1, COL4A1, and PAI-1 were already decreased by 50%, 30%, and 60%, respectively. Further, during the time course when *PVT1* expression was decreased 60–85%, there was a 40–60% decrease in secreted FN1, PAI-1, and COL4A1, but only a 20% reduction in TGFB1. It is reasonable to expect that if the effect of *PVT1* were mediated only by TGFB1, then a correspondingly low decrease (i.e., 20%) in secreted FN1, COL4A1, and PAI-1 would be observed. These findings suggest that *PVT1* may regulate expression of ECM proteins in a manner that is, at least in part, independent of TGFB1 (Fig. 5). Delineation of the relationship between TGFB1 and *PVT1* therefore represents a critical component toward understanding the molecular mechanisms underlying the regulation of ECM in diabetic nephropathy.

In addition to these findings, we also observed effects of high glucose treatment on expression of ECM-related factors in mesangial cells. Although increased secretion of FN1, COL4A1, TGFB1 and PAI-1 has been previously noted [22], [23], [24], [25], there are substantial disparities in the literature regarding the timing and magnitude of the increase in secreted ECM-related proteins by MC following glucose induction (for example, see [22], [25], [26], [27], [28], [29]). Differences among studies may reflect the utilization of different cell lines, cell culture media components, and concentrations of glucose used for induction, as well as the level of sensitivity and specificity associated with each of the different assay methods employed. Considering the discrepancies found in the literature, we therefore sought to characterize the quantification of ECM accumulation in response to glucose in the particular cell line and conditions comprised in this study. Interestingly, we found significant increases in levels of secreted FN1 and PAI-1 at 48 h, 72 h and 96 h (Fig. 1B and 2C), as well as COL4A1 and TGFB1 at 72 h and 96 h (Fig. 1C and 2B), which contrasts with results reported by Wahab et al. [28] and Tada et al. [27], who did not detect increased levels of collagen type IV or PAI-1, respectively, following exposure of primary human MC to HG. However, our results are in agreement with Isono et al. [30], who found a 20% increase in secreted TGFB1 at 72 h in human MC. Interestingly, Oh et al. [26] reported an early increase in *TGFB1* mRNA at 6 h in rat MC under HG conditions, which was not observed in humans, and may reflect a species-specific expression response. Although there is ample discrepancy in expression patterns of FN1, COL4A1, PAI-1, and TGFB1 in MC in response to HG among different reports, for the current study, we are confident that the methods utilized here, TaqMan qPCR and sandwich ELISA, are among the most sensitive and specific to quantify mRNA and proteins, respectively, thus, ensuring the accuracy of our findings.

To date, most investigations of *PVT1* have been conducted in respect to cancer, particularly lymphomas and breast/ovarian tumors, and little is known of the role of *PVT1* in normal tissues, In humans, *PVT1* has been shown to play a role in cell proliferation and apoptosis, and it is likely that the tumorigenic properties of *PVT1* are manifested, at least in part, through this activity. How might *PVT1*, a gene that does not appear to encode a protein product, contribute to the dysregulation of ECM production? One possibility is through the actions of small, non-coding microRNA molecules (miRNA). miRNAs are short, non-coding RNAs that typically bind the 3′-UTR of target mRNAs, leading to posttranscriptional silencing and translational repression [31], [32], [33], [34], RNA degradation [35], [36], [37], or transcriptional inhibition [38]. miRNAs have been widely studied in cancer, where they can act as oncogenes or tumor suppressors [39], [40], but they have also been associated with the regulation of genes involved with insulin secretion, cholesterol biosynthesis, fat metabolism, and adipogenesis [41], [42], [43]. Importantly, miRNAs have been shown to mediate TGFB signaling in diabetic nephropathy, and several candidates, including miR-92, miR-192, miR-216a, miR-217, and miR-377 are upregulated in mesangial cells in response to either glucose or TGFB, and are also indirectly correlated with increased collagen and fibronectin expression [44], [45], [46], [47]. For example, Wang et al. [47] demonstrated that miR-377 leads to reduced expressions of p21-activated kinase and superoxide dismutase, which enhanced fibronectin protein production. Thus, overexpression of miR-377 in diabetic nephropathy indirectly leads to increased fibronectin protein production.

There are at least 6 known miRNAs mapping to the *PVT1* locus, including miR-1204, -1205, -1206, -1207-5p, 1207-3p, and -1208 [48], and it is possible that some of the effects of *PVT1* on ECM factors may be mediated through a miRNA mechanism. Whether *PVT1*-derived miRNAs act in concert with, or independent from, those previously identified miRNAs remains to be seen; however, our preliminary findings suggest that miR-1205, miR-1207-3p, miR-1207-5p, and miR-1208 are up-regulated by high glucose in mesangial cells (data not shown). Additional characterization of *PVT1*-derived miRNAs will be required to fully delineate the relationship between *PVT1* and changes in ECM-related proteins.

Despite the evidence showing involvement of *PVT1* in ECM regulation, we acknowledge several issues that may limit the interpretation of our findings. First, the experiments presented here were performed in primary human mesangial cells and thus, conclusions drawn from the results presented here are necessarily limited to this cell type. We chose to initiate the functional characterization of *PVT1* in this cell type because one of the key hallmarks of diabetic nephropathy is expansion of mesangium. Mesangial cells are directly involved in the expansion of the mesangium, which is comprised of mesangial cells and extracellular matrix, and the extent to which we could pinpoint a role for *PVT1* in this process would determine the direction in which further studies could be designed. However, we recognize that *PVT1* may play a general role among different cell types, including podocytes, renal proximal tubule cells, and others.

Another limitation is our focus on the effect of *PVT1* on ECM-related processes, although this gene may likely be involved in other biological pathways, including cell proliferation and apoptosis (67). *PVT1* may also regulate other ECM components and key ECM regulators not addressed in this study, particularly those detected mainly in late glomerulosclerosis (21), including collagen type I and III, matrix metalloproteinases MMP3, MMP7, MMP10 and MMP1 [49], as well as the proteoglycans, decorin, versican, and perlecan [20]. Given the findings obtained here, the study of additional *PVT1* targets involved with ECM, is well-justified.

In conclusion, the results presented here provide biological support for findings of association with ESRD in individuals with T1D and T2D. We show that *PVT1* is regulated by hyperglycemia, and may mediate susceptibility to diabetic kidney disease through effects involving ECM accumulation. Further characterization of this gene may yield potentially new insight into disease pathogenesis, and additional investigations of the role of PVT1 in the kidney are therefore warranted.

## Methods

### Mesangial cell culture

Primary cultures of normal human mesangial cells (MC) were purchased from Lonza (Walkersville, MA) and cultured in Lonza Mesangial Cell Basal Medium (MsBM) supplemented with 5% fetal bovine serum (FBS) according to the manufacturer's instructions. Briefly, approximately 3500 cells/cm^2^ were seeded in 25 cm^2^ cell culture flasks (Corning Life Sciences; Lowell, MA) containing 5 ml of Lonza Mesangial Cell Basal Medium (MsBM) supplemented with 5% fetal bovine serum (FBS). Cells were placed at 37°C in a Hera Cell 5% CO_2_ incubator (ThermoFisher Scientific; Waltham, MA). Culture medium was replaced the first day after MC seeding, and then again every two days. Cells were subcultured every 7 to 9 days using Clonectis ReagentPack Subculture Reagents (Lonza; Walkersville, MA) according to the manufacturer's instructions. All experiments were performed using cells between the sixth and eighth passage.

### Total RNA extraction and quantification

Total RNA was extracted using the RNeasy Plus Mini Kit (Qiagen; Valencia, CA) according to the manufacturer's instructions. RNA concentration was determined by absorbance at 260 nm and RNA integrity was evaluated using the RNA 6000 Nano Lap Chip Kit; only RNA samples with a RIN>8 and 18S/28S ratio >2.0 were used in the qPCR assays.

### Quantitative real-time RT-PCR (qPCR)

First-strand cDNA was synthesized from total RNA obtained from mesangial cells using random primers and the Super Script III Reverse Transcriptase kit (Invitrogen) according to the manufacturer's protocol. Quantitative real time RT-PCR (qPCR) was performed using commercial TaqMan Gene Expression Assays (Applied Biosystems; Foster City, CA) in conjunction with the ABI Prism 7900 HT Sequence Detector apparatus (Applied Biosystems). Data were normalized using both *PPIA* (cyclophilin A) and *UBC* (ubiquitin C), the 2 most stable housekeeping genes tested for the experimental conditions used. Results were analyzed with RQ Manager software (Applied Biosystems), and qBasePlus v1.5 (Biogazelle NV; Ghent, Belgium). Assay information is provided in the Table S1.

### Protein extraction and quantification

Cell culture media from MC was treated with 0.5 ml absolute ethanol/ml of media for a final 33% v/v ethanol and stored at −20°C for at least 2 hours to precipitate proteins. Samples were then centrifuged at 4,000× *g* for 30 min at 4°C, and the protein precipitate was resuspended in 400 µl of PBS supplemented with Complete Protease Inhibitor Cocktail (Roche; Indianapolis, IN). Total protein content for each sample was determined using the BCA Protein Assay kit (Pierce; Rockford, IL) according to the manufacturer's instructions.

### Enzyme-linked immunosorbent assay analysis (ELISA)

Protein concentration was determined using a commercial sandwich ELISA kit for TGFB1 (R&D Systems; Minneapolis, MN) and PAI-1 (eBiosciences; San Diego, CA), or competitive inhibition ELISA for FN1 (Millipore; Billerica, MA) according to the manufacturer's instructions. COL4A1 was quantified using an indirect ELISA developed in our laboratory. For this assay, total soluble protein was extracted from cell culture media as described above, and extracts were incubated overnight at 4°C in high bind polystyrene EIA 96 wells microplates (Corning Life Sciences), followed by three washes with PBST (PBS plus 0.05% Tween 20), and blocking with 1% bovine serum albumin (BSA) in PBST (blocking buffer) for 2 h at room temperature. After another three washes with PBST, rabbit anti-COL4A1 polyclonal antibody (Abcam; San Francisco, CA) was added at a 1∶1000 dilution in blocking buffer, and incubated for 2 h at room temperature. After another three washes with PBST, the plate was incubated with peroxidase-labeled goat anti-rabbit (Bio-Rad; Hercules, CA) at a 1∶1000 dilution in blocking buffer, and final detection was performed using TMB peroxidase substrate (Bio-Rad). The reaction was stopped after 5 min with 1N H_2_SO_4_, and the optical density was read at 450 nm using a VICTOR_3_ Microplate Reader (Perkin Elmer; Waltham, MA).

### PVT1 knockdown using small interfering RNA (siRNA) in MC

**Figure 6:**
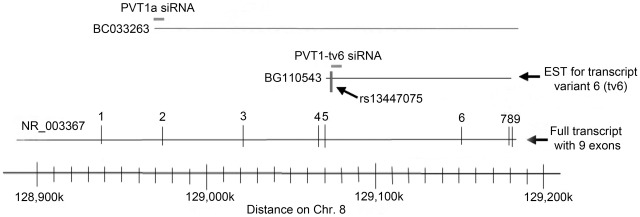
Position of the two *PVT1* siRNAs, PVT1a and PVT1-tv6, used for transfection of mesangial cells. The SNP with the highest association with ESRD in type 1 diabetes, rs13447075, is shown as a vertical line next to the PVT1-tv6 siRNA. Bottom, chromosome distance coordinates in kb from National Center for Biotechnology Information Build 37.1 of the *PVT1* locus.

A mixture of two siRNAs targeting different parts of *PVT1* were used in this study: PVT1a siRNA targets exon 2 and has been shown to knockdown *PVT1* [16], and PVT1-tv6 siRNA (Fig. 6), which was designed from Accession number BG110543 using Dharmacon siDesign (www.dharmacon.com). The target sequence of PVT1-tv6 siRNA was 5′-GCATGGACTTGCAGGCCAA-3′. The EST BG110543 contains marker rs13447075, which was previously found to be associated with ESRD [12]. Approximately 2×10^5^ MC were plated per 25 cm^2^ flask at least 24 h before transfection to achieve 50–70% confluency, then transfected with either 60 nM PVT1a siRNA+PVT1-tv6 siRNA, or negative control siRNA comprised of sequence not found in the human genome (Applied Biosystems; Foster City, CA), using 5 µl Lipofectamine RNAiMax (Invitrogen; Carlsbad, CA) per ml of MsBM media following the manufacturer's instructions. Cells and cell culture media were harvested 48, 72, and 96 h post-transfection for RNA and protein analysis, respectively.

### Statistical analysis

All statistical analyses were performed using the software Graph Pad Prism 5 for Microsoft Windows. One-way ANOVA test with a Dunnett's Multiple Comparison post-test were used to assess differences between conditions. Results were considered statistically significant if *P*<0.05.

## Supporting Information

Table S1 (DOC)

## References

[1] U.S.R.D.S. (2002). United States Renal Data System Annual Data Report: Atlas of End-Stage Renal Disease in the United States.

[2] Jones CAKA, Rogus J, Xue JL, Collins A, Warram JH (2005). Epidemic of end-stage renal disease in people with diabetes in the United States population: Do we know the cause?. Kidney Int.

[3] Nelson RG, Knowler WC, Pettitt DJ, Hanson RL, Bennett PH (1995). Incidence and determinants of elevated urinary albumin excretion in Pima Indians with NIDDM.. Diabetes Care.

[4] Ravid M, Brosh D, Ravid-Safran D, Levy Z, Rachmani R (1998). Main risk factors for nephropathy in type 2 diabetes mellitus are plasma cholesterol levels, mean blood pressure, and hyperglycemia.. Arch Intern Med.

[5] Pettitt DJ, Saad MF, Bennett PH, Nelson RG, Knowler WC (1990). Familial predisposition to renal disease in two generations of Pima Indians with type 2 (non-insulin-dependent) diabetes mellitus.. Diabetologia.

[6] Quinn M, Angelico MC, Warram JH, Krolewski AS (1996). Familial factors determine the development of diabetic nephropathy in patients with IDDM.. Diabetologia.

[7] Seaquist ER, Goetz FC, Rich S, Barbosa J (1989). Familial clustering of diabetic kidney disease. Evidence for genetic susceptibility to diabetic nephropathy.. N Engl J Med.

[8] Craig DW, Millis MP, DiStefano JK (2009). Genome-wide SNP genotyping study using pooled DNA to identify candidate markers mediating susceptibility to end-stage renal disease attributed to Type 1 diabetes.. Diabet Med.

[9] Hanson RL, Craig DW, Millis MP, Yeatts KA, Kobes S (2007). Identification of PVT1 as a Candidate Gene for End-Stage Renal Disease in Type 2 Diabetes Using a Pooling-Based Genome-Wide Single Nucleotide Polymorphism Association Study.. Diabetes.

[10] Pezzolesi MG, Poznik GD, Mychaleckyj JC, Paterson AD, Barati MT (2009). Genome-wide association scan for diabetic nephropathy susceptibility genes in type 1 diabetes.. Diabetes.

[11] Shimazaki A, Kawamura Y, Kanazawa A, Sekine A, Saito S (2005). Genetic Variations in the Gene Encoding ELMO1 Are Associated With Susceptibility to Diabetic Nephropathy.. Diabetes.

[12] Millis MP, Bowen D, Kingsley C, Watanabe RM, Wolford JK (2007). Variants in the plasmacytoma variant translocation gene (PVT1) are associated with end-stage renal disease attributed to type 1 diabetes.. Diabetes.

[13] Graham M, Adams JM (1986). Chromosome 8 breakpoint far 3′ of the c-myc oncogene in a Burkitt's lymphoma 2;8 variant translocation is equivalent to the murine pvt-1 locus.. Embo J.

[14] Mengle-Gaw L, Rabbitts TH (1987). A human chromosome 8 region with abnormalities in B cell, HTLV-I+ T cell and c-myc amplified tumours.. Embo J.

[15] Shtivelman E, Bishop JM (1989). The PVT gene frequently amplifies with MYC in tumor cells.. Mol Cell Biol.

[16] Guan Y, Kuo WL, Stilwell JL, Takano H, Lapuk AV (2007). Amplification of PVT1 contributes to the pathophysiology of ovarian and breast cancer.. Clin Cancer Res.

[17] Ausiello DA, Kreisberg JI, Roy C, Karnovsky MJ (1980). Contraction of cultured rat glomerular cells of apparent mesangial origin after stimulation with angiotensin II and arginine vasopressin.. J Clin Invest.

[18] Blantz RC, Konnen KS, Tucker BJ (1976). Angiotensin II effects upon the glomerular microcirculation and ultrafiltration coefficient of the rat.. J Clin Invest.

[19] Haneda M, Koya D, Isono M, Kikkawa R (2003). Overview of glucose signaling in mesangial cells in diabetic nephropathy.. J Am Soc Nephrol.

[20] Mason RM, Wahab NA (2003). Extracellular matrix metabolism in diabetic nephropathy.. J Am Soc Nephrol.

[21] Steffes MW, Osterby R, Chavers B, Mauer SM (1989). Mesangial expansion as a central mechanism for loss of kidney function in diabetic patients.. Diabetes.

[22] Ayo SH, Radnik RA, Glass WF, Garoni JA, Rampt ER (1991). Increased extracellular matrix synthesis and mRNA in mesangial cells grown in high-glucose medium.. Am J Physiol.

[23] Haneda M, Kikkawa R, Horide N, Togawa M, Koya D (1991). Glucose enhances type IV collagen production in cultured rat glomerular mesangial cells.. Diabetologia.

[24] Hoffman BB, Sharma K, Zhu Y, Ziyadeh FN (1998). Transcriptional activation of transforming growth factor-beta1 in mesangial cell culture by high glucose concentration.. Kidney Int.

[25] Lee EA, Seo JY, Jiang Z, Yu MR, Kwon MK (2005). Reactive oxygen species mediate high glucose-induced plasminogen activator inhibitor-1 up-regulation in mesangial cells and in diabetic kidney.. Kidney Int.

[26] Oh JH, Ha H, Yu MR, Lee HB (1998). Sequential effects of high glucose on mesangial cell transforming growth factor-beta 1 and fibronectin synthesis.. Kidney Int.

[27] Tada H, Tsukamoto M, Ishii H, Isogai S (1994). A high concentration of glucose alters the production of tPA, uPA and PAI-1 antigens from human mesangial cells.. Diabetes Res Clin Pract.

[28] Wahab NA, Harper K, Mason RM (1996). Expression of extracellular matrix molecules in human mesangial cells in response to prolonged hyperglycaemia.. Biochem J.

[29] Wolf G, Sharma K, Chen Y, Ericksen M, Ziyadeh FN (1992). High glucose-induced proliferation in mesangial cells is reversed by autocrine TGF-beta.. Kidney Int.

[30] Isono M, Cruz MC, Chen S, Hong SW, Ziyadeh FN (2000). Extracellular signal-regulated kinase mediates stimulation of TGF-beta1 and matrix by high glucose in mesangial cells.. J Am Soc Nephrol.

[31] Chendrimada TP, Finn KJ, Ji X, Baillat D, Gregory RI (2007). MicroRNA silencing through RISC recruitment of eIF6.. Nature.

[32] Kiriakidou M, Tan GS, Lamprinaki S, De Planell-Saguer M, Nelson PT (2007). An mRNA m7G cap binding-like motif within human Ago2 represses translation.. Cell.

[33] Maroney PA, Yu Y, Fisher J, Nilsen TW (2006). Evidence that microRNAs are associated with translating messenger RNAs in human cells.. Nat Struct Mol Biol.

[34] Petersen CP, Bordeleau ME, Pelletier J, Sharp PA (2006). Short RNAs repress translation after initiation in mammalian cells.. Mol Cell.

[35] Liu J, Valencia-Sanchez MA, Hannon GJ, Parker R (2005). MicroRNA-dependent localization of targeted mRNAs to mammalian P-bodies.. Nat Cell Biol.

[36] Rossi JJ (2005). RNAi and the P-body connection.. Nat Cell Biol.

[37] Sen GL, Blau HM (2005). Argonaute 2/RISC resides in sites of mammalian mRNA decay known as cytoplasmic bodies.. Nat Cell Biol.

[38] Kim DH, Saetrom P, Snove O, Rossi JJ (2008). MicroRNA-directed transcriptional gene silencing in mammalian cells.. Proc Natl Acad Sci U S A.

[39] Bartel DP (2009). MicroRNAs: target recognition and regulatory functions.. Cell.

[40] Kim VN, Han J, Siomi MC (2009). Biogenesis of small RNAs in animals.. Nat Rev Mol Cell Biol.

[41] Heneghan HM, Miller N, Kerin MJ (2010). Role of microRNAs in obesity and the metabolic syndrome.. Obes Rev.

[42] Poy MN, Eliasson L, Krutzfeldt J, Kuwajima S, Ma X (2004). A pancreatic islet-specific microRNA regulates insulin secretion.. Nature.

[43] Poy MN, Spranger M, Stoffel M (2007). microRNAs and the regulation of glucose and lipid metabolism.. Diabetes Obes Metab.

[44] Kato M, Arce L, Natarajan R (2009). MicroRNAs and their role in progressive kidney diseases.. Clin J Am Soc Nephrol.

[45] Kato M, Putta S, Wang M, Yuan H, Lanting L (2009). TGF-beta activates Akt kinase through a microRNA-dependent amplifying circuit targeting PTEN.. Nat Cell Biol.

[46] Kato M, Zhang J, Wang M, Lanting L, Yuan H (2007). MicroRNA-192 in diabetic kidney glomeruli and its function in TGF-beta-induced collagen expression via inhibition of E-box repressors.. Proc Natl Acad Sci U S A.

[47] Wang Q, Wang Y, Minto AW, Wang J, Shi Q (2008). MicroRNA-377 is up-regulated and can lead to increased fibronectin production in diabetic nephropathy.. Faseb J.

[48] Huppi K, Volfovsky N, Runfola T, Jones TL, Mackiewicz M (2008). The identification of microRNAs in a genomically unstable region of human chromosome 8q24.. Mol Cancer Res.

[49] McLennan SV, Fisher E, Martell SY, Death AK, Williams PF (2000). Effects of glucose on matrix metalloproteinase and plasmin activities in mesangial cells: possible role in diabetic nephropathy.. Kidney Int.

