# Supplementary material for: Functional Characterization of the Plasmacytoma Variant Translocation 1 Gene (PVT1) in Diabetic Nephropathy
Source: PLoS One. 2011 Apr 22;6(4):e18671. doi: 10.1371/journal.pone.0018671 (PMC3081298; doi:10.1371/journal.pone.0018671)
Supplement: Table S1 — (DOC) [file pone.0018671.s001.doc]

| **Gene symbol** | **Gene name** | | **ABI Assay (*)** | | **RefSeq** | | **Assay Location** | | **Exon boundary** | | **Amplicon length (bp)** | |
| --- | --- | --- | --- | --- | --- | --- | --- | --- | --- | --- | --- | --- |
| PVT1 | Pvt1 oncogene (non-protein coding) | Hs01069044_m1 | | NR_003367.1 | | 1374 | | 6-7 | | 83 | |  |
| FN1 | Fibronectin 1 | Hs01549976_m1 | | Multiple | | 1482 | | 8-9 | | 81 | |  |
| COL4A1 | Collagen type IV, alpha 1 | Hs01007469_m1 | | NM_001845.4 | | 4577 | | 48-49 | | 87 | |  |
| TGFβ1 | Transforming growth factor, beta 1 | Hs99999918_m1 | | NM_00660.4 | | 1598 | | 4-5 | | 125 | |  |
| SERPINE1 OR PAI-1 | Serpin peptidase inhibitor, member 1 or plasminogen activator inhibitor, type 1 | Hs00167155_m1 | | NM_001165413.1 | | 371 | | 3-4 | | 82 | |  |
| UBC | Ubiquitin C | Hs00824723_m1 | | NM_021009.4 | | 445 | | 1-2 | | 71 | |  |
| PPIA | Peptidylprolyl isomerase A (cyclophilin A) | Hs99999904_m1 | | NM_021130.3 | | 436 | | 4-4 | | 98 | |  |

**Table 1**: ABI TaqMan Assays used in real-time quantitative PCR (qPCR)

(*) ABI Assay: inventoried pre-designed TaqMan Gene Expression Assays (Applied Biosystems; Foster City, CA). Sequences of primers and probe are proprietary of Applied Biosystems.
